# Determinants of Arterial Pressure of Oxygen and Carbon Dioxide in Patients Supported by Veno-Arterial ECMO

**DOI:** 10.3390/jcm11175228

**Published:** 2022-09-04

**Authors:** Stefan Andrei, Maxime Nguyen, Vivien Berthoud, Bastian Durand, Valerian Duclos, Marie-Catherine Morgant, Olivier Bouchot, Belaid Bouhemad, Pierre-Grégoire Guinot

**Affiliations:** 1 Department of Anaesthesiology and Critical Care Medicine, Dijon University Medical Centre, F-21000 Dijon, France; 2 Department of Anaesthesiology and Critical Care Medicine, University of Medicine and Pharmacy “Carol Davila”, 020021 Bucharest, Romania; 3 LNC UMR1231, University of Burgundy and Franche-Comté, F-21000 Dijon, France; 4 Cardiac Surgery Department, Dijon University Hospital, F-21000 Dijon, France

**Keywords:** ECMO, blood gases, determinants, oxygenation, carbon dioxide clearance, parameters

## Abstract

**Background:** The present study aimed to assess the determinants of arterial partial pressure of oxygen (PaO_2_) and carbon dioxide (PaCO_2_) in the early phase of veno-arterial extracorporeal membrane oxygenation (VA ECMO) support. Even though the guidelines considered both the risks of hypoxemia and hyperoxemia during ECMO support, there are a lack of data concerning the patients supported by VA ECMO. **Methods:** This is a retrospective, monocentric, observational cohort study in a university-affiliated cardiac intensive care unit. Hemodynamic parameters, ECMO parameters, ventilator settings, and blood gas analyses were collected at several time points during the first 48 h of VA ECMO support. For each timepoint, the blood samples were drawn simultaneously from the right radial artery catheter, VA ECMO venous line (before the oxygenator), and from VA ECMO arterial line (after the oxygenator). Univariate followed by multivariate mixed-model analyses were performed for longitudinal data analyses. **Results:** Forty-five patients with femoro-femoral peripheral VA ECMO were included. In multivariate analysis, the patients’ PaO_2_ was independently associated with Q_EC_, F_D_O_2_, and time of measurement. The patients’ PaCO_2_ was associated with the sweep rate flow and the P_pre_CO_2_. **Conclusions:** During acute VA ECMO support, the main determinants of patient oxygenation are determined by VA ECMO parameters.

## 1. Introduction

The use of veno-arterial extracorporeal membrane oxygenation (VA ECMO) has improved refractory cardiogenic shock mortality [1,2]. VA ECMO is used for bridge-to-recovery, bridge-to-transplantation, or bridge-to-decision purposes [3,4]. Several recommendations have been published relative to the indications, management and weaning of VA ECMO [5]. However, at bedside, physicians still have to manage the challenges related to interactions between the retrograde flow of the extracorporeal circuit and the native homeostatic systems [6,7,8,9,10]. Some important questions are still not addressed.

Firstly, the determinants of patient’s blood gases (PaO_2_ and PaCO_2_) have never been evaluated in patients on VA ECMO support. Several studies have evaluated the determinants of oxygenation and carbon dioxide clearance in the setting of veno-venous ECMO (VV ECMO), as it is used for respiratory indications [11,12,13,14]. Nevertheless, no study has ever been performed in patients supported by VA ECMO. An extrapolation of VV ECMO to VA ECMO is possible, but it might not reflect the hemodynamic effect of the retrograde aortic flow, particularly with commonly used femoral canulation [6].

Secondly, this hemodynamic phenomenon brings into question whether the oxygenated VA ECMO flow can reach the proximal aortic arch during the first hours of support, altering the difference between the P_post_O_2_and theright radial artery PaO_2_ (delta-PaO_2_). A clear answer is difficult, considering that the maintaining of an optimal equilibrium between VA ECMO and the patient’s native heart and lung function is challenging, according to the medical conditions evolution and illness phase [9].

Recently, the Extracorporeal Life Support Organization (ELSO) published guidelines that consider the risks of both hypoxemia and hyperoxemia during ECMO support [15]. The ELSO experts recommend having a P_post_O_2_ value of 150 mmHg to counterbalance the risk of hyperoxemia and to minimize the risk of hypoxemia [15]. The ELSO guidelines suggest targeting post membrane-normocapnia, but without providing a cut-off value [15]. A review of the published studies found out that these recommendations were proposed without clear published data [16]. In the absence of published data, a further question would be if the P_post_O_2_ cutoff value of 150 mmHg proposed by ELSO guidelines can prevent hyperoxemia or hypoxemia.

The present study was designed to assess the determinants of blood gases (PaO_2_ and PaCO_2_) and determinants of and delta-PaO_2_ in patients supported by VA ECMO. The secondary aim was to evaluate the concordance with the actual ELSO guidelines.

## 2. Materials and Methods

### 2.1. Study Design

We performed a retrospective, observational, single-center study in a university-affiliated cardiac intensive care unit (ICU). Because all data were collected retrospectively from the standard medical chart, this study was not considered as involving human participants (according to French law). Nevertheless, this study was approved by the institutional review board (Ethics Committee of the French Society of Anaesthesia and Critical Care, IRB10252018179). Inclusion criteria were: (i) patients supported by peripheral femoro-femoral VA ECMO, (ii) right radial arterial cannula, and (iii) available pre/post membrane blood gas measurements. We excluded (i) pediatric patients and (ii) patients with femoral arterial catheter for invasive blood pressure monitoring. The patients with femoral arterial catheter for invasive blood pressure monitoring were excluded in order to have a homogenous cohort with the same site for arterial blood measurements.

### 2.2. Patients’ Management

All patients underwent femoro-femoral peripheral VA ECMO. The indications, protocols for implementation, VA ECMO management, and patient management have been described elsewhere [17,18,19]. Briefly, the VA ECMO circuit consists of a polymethylpentene hollow-fiber oxygenator (Maquet oxygenator or EOS Livanova), a magnetically levitated centrifugal pump, and heparin-coated tubing. The VA ECMO circuit is primed with isotonic saline solution. A dose of unfractionated heparin is administered in the absence of contraindications before VA ECMO implantation. The initial ECMO flow rate (Q_EC_) is gradually increased until it corresponds to a cardiac index above 2.5 L min^−1^ m^−2^. Then, Q_EC_ is adapted to blood pressure, arterial lactate clearance, echocardiographic parameters, native heart function, and arterio-venous CO_2_ difference [20]. The fraction of inspired oxygen is progressively increased as needed to prevent ischemia-reperfusion injury. Sweep gas flow rate is initially placed at a ratio of 1:1 to the Q_EC_, and then adjusted according to blood gases. All patients were mechanically ventilated during the study period. Patients were ventilated in the controlled volume mode with tidal volume of 5 to 8 mL kg^−1^ (predicted ideal body weight), respiratory rate between 5 and 12 respiratory rate per minute, and positive end-expiratory pressure (PEEP) between 5 and 12 cm H_2_O, in order to obtain plateau pressure below 25 cm H_2_O. All patients were continuously administered unfractionated heparin 4 h after VA ECMO initiation. The heparin dose was adjusted at least twice daily according to heparinemia, targeting an anti-factor Xa activity between 0.15 and 0.3 UI mL^−1^. Packed red blood cells were transfused to maintain hemoglobin level between 8 and 10 g dL^−1^.

### 2.3. Data Collection

All of the data were collected from the patient medical charts. These included demographic data, comorbidities, medical aetiology, circulatory parameters (systolic, mean and diastolic arterial pressure, heart rate, arterial line pulsatility), VA ECMO parameters (oxygen fraction (F_D_O_2_), sweep flow rate), ventilator settings (FiO_2_, minute volume, PEEP), vasopressor and inotropic therapy, blood gas analysis, ICU length of stay, hospital length of stay, and death. Blood gas analysis was noted at baseline (immediately after ECMO initiation), and at 12 h, 24 h, and 48 h after the initiation of VA ECMO support. For each time point, the blood samples were drawn simultaneously from the radial artery catheter, from the VA ECMO venous line (before the oxygenator), and from the VA ECMO arterial line (after the oxygenator). The arterial line pulsatility was defined as a pulse pressure ≥ 20 mmHg [21]. We calculated the gradient between P_post_O_2_ and radial artery partial pressure (delta-PaO_2_) as the absolute difference between P_post_O_2_ and patient’s radial artery PaO_2_.

### 2.4. Statistical Analyses

Continuous variables were expressed as medians with [25–75%] interquartile range (IQR), or as means ± standard deviation (SD), as appropriate. Categorical variables were expressed as numbers (percentage).

The determinants of PaO_2_, PaCO_2_, and delta-PaO_2_ were evaluated using a mixed effects generalized linear model approach. A random “subject” effect was used in these longitudinal analyses. Firstly, each of the collected variables of interest was introduced as fixed effect variable in a univariate mixed effect model with patient artery measured PaO_2_, PaCO_2_, or delta-PaO_2_, as dependent variables. Secondly, the statistically significant and clinically pertinent variables in univariate analysis were included in multivariate mixed effects generalized linear model models with patient artery measured PaO_2_, PaCO_2_, or delta-PaO_2_, as dependent variables.

The concordance with ELSO guidelines was evaluated based on the blood determinations of post-membrane PO_2_, because the ELSO experts recommend having a P_post_O_2_ value of 150 mmHg and did not provide any cut-off value for post-membrane PCO_2_. Based on the P_post_O_2_ the determinations were classified as ELSO concordant (<150 mmHg) or not. Furthermore, the patients’ PaO_2_ right radial artery determinations were classified as hyperoxemic (>120 mmHg) or as hypoxemic (<70 mmHg). The association the ELSO concordance (yes/no), hyperoxemia (yes/no), or hypoxemia (yes/no) was evaluated using an exploratory logistical regression model.

The statistical analyses were performed using RStudio (Version 1.1.447—© 2022–2018 RStudio, Inc. RStudio: Integrated Development for R. RStudio, PBC, Boston, MA, USA). The threshold for statistical significance was set at *p* < 0.05.

## 3. Results

The patients’ characteristics are shown in Table 1.

Forty-five patients were included in the study analyses. The mean age was 59 ± 12 years, and they were mostly male (77%), with a baseline SOFA score of 13 ± 2. The main indications for VA ECMO support were cardiopulmonary resuscitation (CPR), post-acute myocardial infarction cardiogenic shock, and post-cardiotomy cardiogenic shock. The description of patients’ hemodynamic and respiratory characteristics at each timepoint of the study is presented in Supplementary Table S1.

### 3.1. Determinants of Arterial Partial Pressure of Oxygen (PaO_2_)

The univariate analyses found that the patients’ PaO_2_ was associated with the ventilator FiO_2_, Q_EC_, sweep rate, F_D_O_2_, pre-ECMO-membrane pH and post-ECMO-membrane pH, PaO_2_, and HCO_3_. The multivariate analysis demonstrated that Q_EC_, the F_D_O_2_, and the time point of the measurement were the only variables independently associated with patient PaO_2_ (Table 2).

### 3.2. Determinants of Arterial Partial Pressure of Dioxide Carbon (PaCO_2_)

In univariate analyses we found that the patients’ PaCO_2_ was associated with pre- and post-membrane pressure of gases, but not with the ventilatory settings. In multivariate analysis, the sweep rate flow and the P_pre_CO_2_ were independently associated with patients’ PaCO_2_ (Table 3). Ventilatory setting were not associated with patients’ PaCO_2_.

### 3.3. Determinants of Delta-PaO_2_ (Difference between Post-Membrane PO_2_ and PaO_2_)

The median delta-PaO_2_ was 11 [−14–67] mmHg. In univariate analyses, delta-PaO_2_ was associated with arterial line pulsatility, pre-membrane pH, S_pre_O_2_, P_pre_O_2_, P_pre_CO_2_, post-membrane pH, P_post_O_2_, P_post_CO_2_, S_post_O_2_, and F_D_O_2_. The multivariate analysis demonstrated that F_D_O_2_ and arterial pulsatility were significantly associated with delta-PaO_2_ (Supplementary Table S2).

The timing of measurement was not independently associated with the delta-PaO_2_.

### 3.4. Accordance with ELSO Guidelines

Fifty-nine (33%) blood determinations of P_post_O_2_ were adequate (<150 mmHg), according to the recent ELSO recommendations. Of these 59 determinations, 22 (37%) had a radial artery PaO_2_ over 120 mmHg (hyperoxemia) and 14 (24%) a radial artery PaO_2_ lower than 70 mmHg (hypoxemia).

In all cohort, the concordance with ELSO guidelines was protective of patient hyperoxemia (OR 0.15, 95%CI 0.01-0.31, *p* < 0.001), but it was also associated with a risk of patient hypoxemia (OR 6.78, 95%CI 2.1–21.8, *p* = 0.001).

## 4. Discussion

Our findings can be summarized as following: (i) VA ECMO settings were the main contributor to patient oxygenation and CO_2_ removal during the first 48 h of support, (ii) the ventilator parameters had no significant clinical effect on oxygenation or CO_2_ removal during the acute phase of VA ECMO support, (iii) a patient’s pulsatile arterial flow pattern was associated with higher delta-PaO_2_, and (iv) maintaining a P_post_O_2_ lower than 150 mmHg can be associated with an increased risk of hypoxemia.

Our study demonstrated that VA ECMO oxygenation parameters, and particularly ECMO membrane oxygenation parameters (F_D_O_2_, sweep flow rate), are the main determinants of patients’ PaO_2_. These results are similar to those demonstrated in VV ECMO, where patients’ oxygenation was dependent on the ratio between VV ECMO Q_EC_ and patient cardiac output, and VV ECMO oxygenation parameters [11]. The importance of VA ECMO PO_2_ might also suggest that the so-called oxygenshed phenomenon is not a very significant concern in the acute phase [22]. Even though we did not specifically measure it, our findings suggest that the retrograde aortic flow of VA ECMO was able to reach the proximal aortic arch (i.e., right radial artery). We did not find an association between hemoglobin and oxygenation, as described by some authors for VV ECMO [12,23,24]. This can be explained by the hemoglobin value, which was not very low in the patients from our cohort, and the Q_EC_ which was high, thus maintaining oxygen delivery [23]. We also found that oxygenation performance of the ECMO membrane decreases over time, even during the relatively short time period of 48 h, and despite anticoagulation. This observation, which confirms a prior study in VV ECMO patients [25], is explained by clot formation and cellular deposit on the fibers of the membrane [25,26].

Similarly to oxygenation, patient’s CO_2_ removal mainly depends on VA ECMO. However, due to CO_2_ different physical properties, there are some notable differences. Interestingly, the P_pre_CO_2_ was independently associated with PaCO_2_. The role of pre-membrane CO_2_ seemed similar in a previous experimental VV ECMO study [13]. This finding underlies the importance of the patient’s metabolic status, even during acute shock. The importance of the sweep gas flow rate has already been described for VV ECMO [11]. The same study and various others have reported that Q_EC_ and FiO_2_ have little effect on CO_2_ removal [11,27], but this is inconsistent with other experimental studies [13]. Contrary to oxygenation, time had no impact on membrane CO_2_ removal, at least during the first 48 h. This finding is explained by the high diffusion coefficient of CO_2_.

The gradient of oxygenation (delta-PaO_2_) between the ECMO P_post_O_2_ and the patient’s PaO_2_ was influenced by ECMO F_D_O_2_ and arterial pulsatility. These results emphasize the main role of ECMO parameters, and particularly the residual cardiac function of the patient. A low pulse pressure during VA reflects a low native cardiac output [28]. Retrograde aortic flow into the aorta during VA ECMO can cause upper body hypoxemia in relation to the competitive flows between the native blood flow and the VA ECMO blood flow (Q_EC_), particularly in the case of the commonly used peripheral femoro-femoral cannulation [29,30,31]. Several mixed blood zones have been observed because the mixed blood zone location depends on native heart and lung functionality and on the hemodynamic support provided by VA ECMO [32]. It is worth noting that 50% of the patients in our study had arterial pulsatility, and that PaO_2_ measured from the radial artery is a sensitive tool that is readily available at bedside to detect oxygenation imbalance [33]. The fact that arterial pulsatility was positively associated with the gradient of oxygenation between the ECMO membrane in our cohort reflects the effect of cardiac function on the oxygenation of the higher body part. Thus, this gradient could be analyzed as a variable reflecting the ratio between native cardiac/lung function and VA ECMO retrograde flow in the oxygenation of the body. In case of a pulsatility index over 20 mmHg, physicians should be aware of the risk of the oxygenshed phenomenon, but further studies are needed to confirm this point.

In our study, only one third of the P_post_O_2_ determinations were in concordance with the ELSO cut-off of <150 mmHg. This rather low concordance level might be explained by the fact that the patients were managed before the issue of this recommendations. The ELSO concordance was associated with patients’ hypoxemia. Our cohort limited size did not allow for a complex analysis including all the possible confounding factors. The association with patient’s hypoxemia might be simply due a more severe hemodynamic status. However, this result confirms the need for large trials on this topic.

### 4.1. Clinical Implications

This study provided the first data of the determinants of patients’ blood gases during acute VA ECMO support, answering a both hemodynamic and ventilatory question. During the acute phase of circulatory VA ECMO support, patient oxygenation and CO_2_ removal is often overlooked. However, these factors are of importance for several reasons. Both hyperoxemia and hypoxemia are associated with worse outcomes [34]. Moreover, changes in PaCO_2_ levels affect neurological outcomes [35]. In VA ECMO-supported patients, blood oxygenation and CO_2_ removal are difficult to predict at bedside. Our results reflect real life for many patients who were hyperoxemic or hypoxemic.

When implementing VA ECMO, the ECMO team should be aware of the parameters of the VA ECMO sweep gas blender setting in regard to Q_EC_. Hyperoxia affects outcomes and mortality. F_D_O_2_ should, therefore, be probably started at 0.6 because the probability of hypoxemia is low at this setting, since the PaO_2_ is over 100 mmHg in most cases [35], and because Q_EC_ is higher than native cardiac output. Because sweep gas flow is the main determinant of patient PaCO_2_, it should be carefully adapted to PaCO_2_ and to patient metabolic status. In our cohort, the median PaCO_2_ was low, with a ratio of sweep gas flow to Q_EC_ that was probably too high. We observed slow changes in sweep gas flow, and PaCO_2_ changes were within acceptable values. At the implantation of VA ECMO, sweep gas flow should probably not be too high and could be set at a ratio of 0.7 to Q_EC_. Considering both our results and a priori data, physicians should probably measure blood gases as early as possible to adapt VA ECMO sweep gas blender setting parameters.

None of the ventilator parameters were associated with oxygenation or CO_2_ removal because Q_EC_ was high, and up to 50% of patients had very low cardiac output with no blood pulsatility. In this context, physicians should focus on the parameters of the VA ECMO to avoid anomalies in oxygenation and CO_2_ removal. Ventilator parameters, such as respiratory rate, ventilatory FiO_2_, or PEEP, were not associated with patient homeostasis. Nevertheless, studies have demonstrated that ventilatory parameters should be set to avoid lung trauma [36]. In other words, physicians should consider using protective ventilation with low FiO_2_ and low respiratory rate in patients supported by VA ECMO without any concerns about patient oxygenation or CO_2_ removal. This decision should be taken also based on the delta-PaO_2_ and the pulsatility that indicate the cardiac contribution to the systemic circulation.

This approach becomes even more important during the weaning process, where the decrease in Q_EC_ and the restoration of native cardiac blood flow through pulmonary circulation may favor competitive flow, and thus, local hypoxemia and tissue hypoperfusion [37]. In this context, the gradient between P_post_O_2_ and patient’s PaO_2_ (delta-PaO_2_) could provide information on competing flow. In addition, most patients supported by VA ECMO undergo early extubation [17]. In this case, clinical examination and pulse oximetry may help to detect hypoxemia and/or “harlequin syndrome” during the decrease in VA ECMO support and the weaning process. Furthermore, in light of the recent ELSO recommendations preventing deleterious hypoxemia, the clinician should also be aware of the risk of patients’ hypoxemia.

### 4.2. Limits

The study is limited by its retrospective, observational, exploratory design, and causality cannot be inferred. Considering the high complexity of the ICU clinical situations requiring VA ECMO support, our study design is limited. We included only patients on femoro-femoral VA ECMO. Some important cardio-respiratory parameters were not available for the analysis, such as the native cardiac output for all patients or the native respiratory function and pulmonary shunt [27]. Furthermore, we did not evaluate middle and long-term lung function or patient outcomes. Best ventilation parameters was not an objective of the study [38]. These results are limited to the acute phase (i.e., the first 48 h) and cannot be extrapolated to later phases. However, the acute phase is highly critical because association between hyperoxemia, hypoxia, CO_2_ removal, and clinical outcomes has been demonstrated [39,40].

## 5. Conclusions

During the early VA ECMO support, the main determinants of patients’ oxygenation and carbon dioxide removal are the parameters the VA ECMO. Q_EC_ and F_D_O_2_ are associated with patient oxygenation, whereas sweep gas flow and P_pre_CO_2_ are associated with PaCO_2_. Further studies in patients with varying Q_EC_ and during the weaning process may lead to a better understanding of the interactions between Q_EC_ and native flow.

## Figures and Tables

**Table 1:** Patients’ demographic and hemodynamic characteristics at baseline.

Variables	All Cohort (*n* = 45)
Age (years), mean ± SD	58 ± 11
Female gender, *n* (%)	10 (22)
BMI (kg m^−2^), mean ± SD	28.2 ± 5
SOFA score, mean ± SD	13 ± 2
SAPS 2 score, mean ± SD	69 ± 22
Pulsatility (yes), *n* (%)	21 (47)
Vasoactive and inotropic agents	
Norepinephrine, *n* (%)	29 (64)
Dobutamine, *n* (%)	12 (27)
Epinephrine, *n* (%)	26 (58)
Indication for VA-ECMO, *n* (%)	
Cardiac Arrest	12 (27)
Post cardiotomy shock	15 (33)
Medical cardiogenic shock	15 (34)
Drug intoxication	3 (7)
ECMO baseline parameters	
- Q_EC_ (L min^−1^), median [IQR]	4.1 [3.7–4.8]
- Sweep rate (L min^−1^), median [IQR]	4.5 [4–5.4]
- F_D_O_2_ (%), median [IQR]	80 [70–100]
Ventilatory parameters	
- FiO_2_ (%), median [IQR]	60 [50–100]
- Respiratory rate (min^−1^)	14 [12–16]
- Tidal volume (mL Kg^−1^), mean ± SD	5.8 [5.1–6.4]
- PEEP, median [IQR]	5 [5,6]
28-day mortality, *n* (%)	28 (62)

Abbreviations: BMI—body mass index; SOFA—sequential organ failure assessment; SAPS—simplified acute physiologic score; ECMO—extracorporeal membrane oxygenation; VA—veno-arterial; FiO_2_—fraction of inspired oxygen; F_D_O_2_—fraction of membrane oxygen; PEEP—positive end-expiratory pressure; SD—standard deviation; IQR—interquartile range.

**Table 2:** Mixed generalized linear model with random “subject” for longitudinal analysis of the variables associated with right radial artery PaO_2_. A univariate analysis was followed by a multivariate analysis.

Variables	Univariate Model			Multivariate Model		
	Estimate	Std Error	*p*-Value	Estimate	Std Error	*p*-Value
Norepinephrine (yes)	−20.2	18.1	0.267			
Dobutamine (yes)	−23	18.5	0.216			
Epinephrine (yes)	21	19	0.273			
Pulsatility (yes)	−6.3	16.6	0.702			
Hemoglobin (g dL^−1^)	−6.5	4	0.112			
*Ventilatory parameters*						
FiO_2_ (%)	0.99	0.47	0.037	0.33	0.44	0.442
Minute volume (mL kg^−1^)	0.21	0.42	0.614			
PEEP (cmH_2_0)	−6	4.2	0.151			
*ECMO parameters*						
Q_EC_	−23.8	10.2	0.020	−23.6	9.87	0.018
% of theoretical flow	−0.97	0.45	0.03			
Sweep rate	14.5	5.4	0.007	5.11	5.44	0.348
F_D_O_2_	3.1	0.47	<0.001	2.33	0.52	<0.001
*Pre-membrane blood gases*						
pH	−224	59	<0.001	−238	160	0.139
PO_2_	0.26	0.23	0.267			
PCO_2_	−0.05	1	0.957			
HCO_3−_	−0.37	0.6	0.539			
Blood Saturation	1.1	0.54	0.04	0.89	0.49	0.069
*Post-membrane blood gases*						
pH	−188	62	0.002	45.4	165	0.784
PO_2_	0.56	0.06	<0.001			
PCO_2_	−1.3	1.09	0.245			
HCO_3−_	−5.4	1.8	0.003	1.19	2.19	0.588
Blood saturation (%)	1.8	1.07	0.093			
Time point of measurement				−20.5	7.6	0.007

Abbreviations: ECMO—extracorporeal membrane oxygenation; VA—veno-arterial; FiO_2_—fraction of inspired oxygen; F_D_O_2_—fraction of membrane oxygen; PEEP—positive end-expiratory pressure.

**Table 3:** Mixed generalized linear model with random “subject” for longitudinal analysis of the variables associated with right radial artery PaCO_2_. A univariate analysis was followed by a multivariate analysis.

Variables	Univariate Model			Multivariate Model		
	Estimate	Std Error	*p*-Value	Estimate	Std Error	*p*-Value
Norepinephrine (yes)	−0.19	1.37	0.892			
Dobutamine (yes)	−1.89	1.42	0.185			
Epinephrine (yes)	1.71	1.43	0.237			
Pulsatility (yes)	−2.13	1.24	0.087			
Hemoglobin (g dL^−1^)	−0.22	0.3	0.45			
*Ventilatory parameters*						
FiO_2_ (%)	−0.06	0.03	0.11			
Minute volume (mL kg^−1^)	0.002	0.02	0.93			
PEEP (cmH_2_0)	0.01	0.31	0.966			
*VA ECMO parameters*						
Q_EC_	0.61	0.78	0.439			
Sweep rate flow	0.5	0.41	0.227	0.66	0.28	0.022
F_D_O_2_	1.45	0.04	0.833			
*Pre-membrane blood gases*						
pH	−15	4.5	0.001	9.27	9.3	0.322
PO_2_	−0.008	0.01	0.63			
PCO_2_	0.66	0.05	<0.001	0.72	0.06	<0.001
HCO_3−_	0.005	0.04	0.895			
Blood saturation	−0.09	0.04	0.03	0.06	0.03	0.043
*Post-membrane blood gases*						
pH	−16.7	4.6	<0.001	−5	9.44	0.597
PO_2_	−0.01	0.005	0.008	−0.006	0.003	0.078
PCO_2_	0.72	0.057	<0.001			
HCO_3−_	0.17	0.14	0.224			
Blood saturation	−0.04	0.08	0.552			
Time point of measurement				−0.3	0.4	0.448

Abbreviations: ECMO—extracorporeal membrane oxygenation; VA—veno-arterial; FiO_2_—fraction of inspired oxygen; F_D_O_2_—fraction of membrane oxygen; PEEP—positive end-expiratory pressure.

## Data Availability

Data are available upon reasonable request upon the corresponding author.

## Supplementary Materials

The following supporting information can be downloaded at: https://www.mdpi.com/article/10.3390/jcm11175228/s1, Table S1: Patients’ characteristics at each timepoint (baseline, H12, H24, and H48); Table S2: Mixed generalized linear model with a random “subject” for longitudinal analysis of the variables associated with delta-PaO_2_ (the difference between P_post_O_2_–and patient’s right radial artery PaO_2_). A univariate analysis was followed by a multivariate analysis.
