# Supplementary material for: Determinants of Arterial Pressure of Oxygen and Carbon Dioxide in Patients Supported by Veno-Arterial ECMO
Source: J Clin Med. 2022 Sep 4;11(17):5228. doi: 10.3390/jcm11175228 (PMC9457238; doi:10.3390/jcm11175228)
Supplement: Supplementary file 1 [file jcm-11-05228-s001.zip › jcm-1779841-supplementary.pdf]

Supplementary material legend

Supplementary Table S1. Patients' characteristics at each timepoint (baseline, H12, H24 and H48).

Supplementary Table S2**.** Mixed generalized linear model with a random "subject" for longitudinal analysis of the variables associated with delta-PaO2 (the difference between $P_{post}O_2$ and patient's right radial artery PaO_2). A univariate analysis was followed by a multivariate analysis.

Supplementary Table S1. Patients' characteristics at each timepoint (baseline, H12, H24 and H48).

Variables	Baseline	H12	H24	H48
MAP (mmHg), median [IQR]	74 [66-81]	74 [65-84]	72 [65-87]	75 [65-92]
Pulsatility (yes), n (%)	21 (47)	18 (40)	21 (47)	20 (44)
HR, median [IQR]	92 [70-109]	92 [75-104]	89 [70-99]	90 [67-95]
Norepinephrine (yes), n (%)	29 (64)	26 (58)	27 (60)	23 (51)
Dobutamine (yes), n (%)	12 (27)	14 (31)	18 (40)	14 (31)
Epinephrine (yes), n (%)	26 (58)	24 (57)	21 (47)	12 (27)
Haemoglobin (g dL^{-1})	10.6 [9.1-12.5]	10 [9.1-12.5]	10.1 [9-12]	9.7 [8.6-11.5]
Ventilatory parameters				
FiO_2 (%), median [IQR]	60 [50-100]	50 [50-60]	60 [50-70]	55 [50-70]
Tidal volume (mL kg^{-1}) , median [IQR]	5.8 [4.9-6.3]	5.5 [4.9-6]	5.5 [4.8-6.3]	5.3 [4.6-5.8]
PEEP (cmH_20) , median [IQR]	5 [5-6]	5 [5-6]	5 [5-7]	5 [5-7]
Respiratory rate, median [IQR]	14 [12-15]	14 [14-15]	14 [12-16]	12 [10-16]
VA ECMO parameters				
Q_{EC} (L min-1) , median [IQR]	4.1 [3.6-4.8]	3.8 [3.5-4.4]	4 [3.6-4.5]	4.1 [3.7-4.6]
% theoretical cardiac output	86 [76-94]	79 [72-94]	84 [77-98]	84 [78-97]
Sweep rate flow, median [IQR]	4.5 [4-5.8]	4 [4-5.5]	4 [3.5-5]	4 [2.8-5]
F_DO_2, median [IQR]	80 [70-100]	70 [60-80]	70 [65-80]	70 [65-80]
Right radial artery blood gases				
pH, median [IQR]	7.3 [7.17-7.38]	7.28 [7.2-7.39]	7.32 [7.26-7.43]	7.39 [7.3-7.49]
PO_2 (mmHg), median [IQR]	231 [148-327]	125 [91-182]	135 [90-195]	127 [75-177]
PCO_2 (mmHg), median [IQR]	27 [24-33]	33 [28-39]	33 [29-37]	35 [26-38]
HCO_3-, median [IQR]	13.7 [10.6-16.7]	17.1 [13.2-19]	17.6 [15.5-20.1]	19.6 [16.6-21.2]
Blood saturation, median [IQR]	99.3 [98.4-99.7]	98.8 [96.2-99.4]	99 [96.6-99.5]	99 [97.2-99.6]
Pre-membrane blood gases				
pH, median [IQR]	7.27 [7.12-7.34]	7.26 [7.19-7.39]	7.27 [7.2-7.389]	7.34 [7.26-7.4]
PO_2, median [IQR]	54 [43-66]	41 [38-47]	41 [37-48]	40 [36-47]
PCO_2, median [IQR]	3 [31-42]	40 [33-45]	40 [37-46]	43 [39-47]
HCO_3-, median [IQR]	15.1 [11.5-19.3]	18.3 [15.6-20.9]	20.4 [16.8-22.1]	21.9 [19-24.2]
Blood saturation, median [IQR]	77.5 [71-85.8]	71.9 [64.9-79.9]	73.3 [70-80.1]	71.6 [63.4-77.3]
Post-membrane blood gases				
pH, median [IQR]	7.29 [7.16-7.43]	7.28 [7.23-7.4]	7.3 [7.24-7.41]	7.33 [7.3-7.4]
PO_2, median [IQR]	301 [215-386]	155 [92-272]	164 [91-252]	140 [78-220]
PCO_2, median [IQR]	29 [24-33]	33 [28-39]	35 [31-40]	38 [35-41]
HCO_3-, median [IQR]	13.7 [10.8-17.1]	16.7 [13.7-19.5]	18.7 [15.6-20.8]	21 [17.6-22.7]
Blood saturation, median [IQR]	99.6 [99-99.9]	98.9 [96.9-99.7]	99.4 [97.2-99.6]	98.8 [95.9-99.6]

Abbreviations: MAP – mean arterial pressure; HR – heart rate; ECMO- extracorporeal membrane oxygenation; VA - veno-arterial; FiO2 – fraction of inspired oxygen; FmO2 – fraction of membrane oxygen; PEEP – positive end-expiratory pressure;

Supplementary Table S2. Mixed generalized linear model with a random "subject" for longitudinal analysis of the variables associated with delta-PaO2 (the difference between $P_{post}O_2$ and patient's right radial artery PaO_2). A univariate analysis was followed by a multivariate analysis

**Variables**	**Univariate model**			**Multivariate model**		
	Estimate	Std error	*p-value*	Estimate	Std error	*p-value*
Norepinephrine (yes)	-15.7	18	0.383			
Dobutamine (yes)	23	19	0.237			
Epinephrine (yes)	22	20	0.284			
**Pulsatility (yes)**	**33**	**16**	**0.038**	**32.7**	**15.8**	**0.04**
Haemoglobin (g dL^{-1})	2.4	3.8	0.527			
Ventilatory parameters						
FiO_2 (%)	0.76	0.44	0.088			
Minute volume (mL kg^{-1})	-0.36	0.36	0.317			
PEEP (cmH_20)	8.2	4.2	0.052			
VA ECMO parameters						
Q_{EC}	-13.6	9.7	0.164			
Sweep rate flow	-1.4	5.4	0.79			
**F_DO_2**	**1.52**	**0.48**	**0.002**	**1.55**	**0.5**	**0.002**
Pre-membrane blood gases						
pH	145	62	0.02	152	164	0.356
PO_2	-0.44	0.2	0.028	-0.37	0.19	0.061
PCO_2	-1.96	0.92	0.034	0.96	1.8	0.595
HCO_{3-}	-0.5	0.53	0.344			
Blood saturation	1.05	0.48	0.003	0.56	0.5	0.281
Post-membrane blood gases						
pH	152	62	0.015	-33	168	0.844
PO_2	0.44	0.057	<0.001			
PCO_2	-2.9	0.97	0.003	-2.2	1.9	0.257
HCO_{3-}	-0.19	1.82	0.914			
Blood saturation	2.19	0.9	0.018	1.08	0.92	0.242
Timing				-8	6.4	0.213
Abbreviations: ECMO- extracorporeal membrane oxygenation; VA - veno-arterial; FiO_2 – fraction of inspired oxygen; F_DO_2 – fraction of membrane oxygen; PEEP – positive end-expiratory pressure;						
